# Epidemiological Investigation of Meningeal Worm-Induced Mortalities in Small Ruminants and Camelids Over a 19 Year Period

**DOI:** 10.3389/fvets.2022.859028

**Published:** 2022-04-06

**Authors:** Charlena Keane, Katherine M. Marchetto, Luiz Gustavo R. Oliveira-Santos, Arno Wünschmann, Tiffany M. Wolf

**Affiliations:** ^1^Department of Veterinary Population Medicine, University of Minnesota, St. Paul, MN, United States; ^2^Department of Ecology, BioScience Institute, Federal University of Mato Grosso do Sul, Campo Grande, Brazil

**Keywords:** brainworm, *Parelaphostrongylus tenuis*, parelaphostrongylosis, sheep, goat, llama, alpaca, cerebrospinal nematodiasis

## Abstract

Meningeal worm, or *Parelaphostrongylus tenuis* (*P. tenuis*) is a nematode parasite that can invade the nervous system of small ruminant and camelid species such as alpaca, llama, goats and sheep. Limited reports exist on the epidemiology of disease caused by the nematode in susceptible livestock. We examined archived necropsy reports from small ruminant and camelid mortalities that were submitted, post mortem, to the University of Minnesota Veterinary Diagnostic Laboratory (MNVDL) during 2001–2019 for gross necropsy, histopathology, and pathogen screening. We estimated *P. tenuis*-induced mortality over time and developed temporal models to better understand patterns and drivers of *P. tenuis*-induced mortalities in these animals. During the period under examination, 5,617 goats, sheep, llamas and alpacas were necropsied, revealing an overall *P. tenuis*-induced mortality rate of 1.14% in the necropsy submission pool for these species. *P. tenuis*-induced mortality rates were highest in llamas (9.91%) and alpacas (5.33%) compared to sheep and goats (<1%), with rates in llamas and alpacas significantly higher than in sheep and goats. *P. tenuis-*induced mortalities exhibited one seasonal peak, around October to December. *P. tenuis*-induced mortality rates varied greatly between years, and have significantly increased over time. We also observed a positive correlation between summer temperature (range 20.4–22.4°C) and *P. tenuis-*induced mortality rates (range 0–3.9%), but not precipitation. This study demonstrates seasonal patterns and differences in mortality between alpacas, goats, llamas and sheep and helps us to better understand the epidemiology of *P. tenuis* mortality.

## Introduction

*Parelaphostrongylus tenuis* (*P. tenuis*), commonly known as meningeal worm, is a nematode parasite of wildlife that spills over into domestic livestock, causing significant morbidity and mortality. It is transmitted from white-tail deer (*Odocoileus virginianus*) to susceptible ungulate livestock such as alpacas (*Vicugna pacos*), goats (*Capra aegagrus hicus*), sheep (*Ovis aries*), or llamas (*Lama glama*) (1, 2). White-tailed deer are the natural, definitive host in which *P. tenuis* inhabits and reproduces within their central nervous system with rare clinical or population effects. Small ruminants and camelids, hereafter collectively referred to as small ruminants, are aberrant hosts that become infected when they incidentally consume infected intermediate hosts, terrestrial gastropods, while foraging (3, 4). *P. tenuis* infection is a concern for livestock across much of mid to eastern North America where white-tailed deer inhabit. For instance, in a high case year up to 22% of a goat herd may become infected, with 44% mortality of infected individuals without treatment (5). Llama herds have experienced 36% infection rates in a high case year, with a 75% mortality of infected individuals (6).

The complex terrestrial lifecycle of *P. tenuis* begins when L1 larvae are shed in the feces of white-tailed deer (7, 8). The larvae are then picked up by snails and slugs, which act as intermediate hosts. Once in the gastropods, *P. tenuis* larvae develop to the L3 stage and are then ingested by grazing ruminants. The L3 larvae leave the gastropods once in the gastrointestinal tract of the mammalian host and migrate through the body to the spinal cord. In white-tailed deer, the nematode inhabits the subarachnoid space of the meninges where it sexually reproduces. The eggs enter the venous circulation and migrate through capillaries to the lungs. Once in the lungs, the larvae are coughed up, swallowed and then passed through the gastrointestinal tract and excreted with feces (7, 9).

In aberrant hosts, such as small ruminants, the larvae migrate from the gastrointestinal tract into the dorsal horn of the spinal cord, and continue to migrate through the spinal cord and to the brain (7, 9). This aberrant migration causes a condition known as cerebrospinal nematodiasis, which may result in mild to severe neurological disease and possible death of the animal. Definitive diagnosis is made by identifying the nematode in the brain and/or spinal cord during necropsy or histopathology (7) and is otherwise assumed based on a combination of neurological signs and characteristic histological changes associated with nematode migration in the central nervous system (2, 9). Antemortem diagnosis is presumptive based on clinical signs and evidence of a marked increase in eosinophils in the cerebrospinal fluid (if sampled), though identification of the nematode is rare (7, 10).

Given the challenges in diagnosis and the fact that treatment of this disease in small ruminants is not always effective (9), attention has been directed toward reduction of risk related to contact with the intermediate hosts or overlapping landscape with white-tailed deer. Treatment consists of multiple doses of an anthelmintic, often accompanied by anti-inflammatory drugs (11). Environmental risk factors that have been suggested to increase *P. tenuis* transmission by gastropods include increased moisture and temperature, factors which are associated with increased gastropod activity (8). Co-occurrence with deer also increases risk of infection, especially in areas preferred by white-tailed deer during spring, when higher incidence of larval shedding has been reported (8). *P. tenuis* seasonality and particularly yearly variation are poorly reported in domestic livestock, and much of this information is anecdotal (7, 9, 12). Having a better understanding of the epidemiological patterns of disease will help us understand when risk is the highest and help to guide disease management.

Our objective was to examine the patterns of *P. tenuis*-induced mortality over time and to describe the variations in the epidemiology of *P. tenuis* in small ruminants and camelids in and around Minnesota. We accomplished this by reviewing necropsy reports submitted to the University of Minnesota Veterinary Diagnostic Laboratory (MNVDL) between 2001 through 2019. We hypothesized that patterns of *P. tenuis*-induced mortality would elucidate seasonal or weather-related (i.e., temperature and precipitation) risks associated with *P. tenuis* infection. Although not all infected small ruminants die following infection (e.g., given treatment intervention) and mortality lags infection by 28–60 days (12, 13), we assumed trends in mortality would be a reasonable indicator for trends in infection.

## Materials and Methods

### Pathology Case Data

Data were collected from necropsy case reports from the University of Minnesota's Veterinary Diagnostic Lab (MNVDL) from 2001 to 2019. Ninety-three case reports of alpacas, llamas, goats and sheep were identified from a total of 5,617 necropsy records from the MNVDL pathology database for inclusion in the data set based on the search terms “*Parelaphostrongylus tenuis*”, “*P. tenuis*,” and “meningeal worm.”

Case reports were individually screened to determine whether they were a confirmed *P. tenuis* case, a presumptive *P. tenuis* case, or no evidence of *P. tenuis* involvement. Confirmed cases were those where a nematode was found or migration tracts were observed histologically. Presumptive cases were those that did not display direct evidence of *P. tenuis* presence, but were characterized by eosinophilic, randomly distributed lesions described as encephalitis, meningoencephalitis, myelitis, meningomyelitis, encephalomyelitis, or meningoencephalomyelitis. Case reports that were identified in the database search but excluded from further review and analysis were those where *P. tenuis* may have been suspected based on clinical signs leading up to mortality, but infection was ruled out based on absence of consistent histological lesions.

Data were collected from the case reports using pdf scraping in the statistical computing environment R in the manner described below (14), and then manually verified. The R package *pdftools* was used to convert pdf format case reports into searchable plain text (15). The following information was extracted from each case report: accession number, year received, death date, date received, date sampled, species, breed, sex, age, weight, body condition score, owner's location, reproductive state (neutered, intact, or pregnant), clinical signs, and whether *P. tenuis* or related search terms were mentioned in the diagnostic and comments sections of reports. The clinical signs searched for in the case reports and recorded included: neurological signs, ataxia, recumbency or being down, tremors, opisthotonos, and abnormal mentation. Not every case report included the date of death. When this information was not available, the date of carcass submission to the MNVDL was used for analysis of seasonality. In cases where both death date and date received were available, the mean difference between death date and date received was 1.2 days (st. dev. 1.41 days). Thus, we considered it reasonable to substitute submission date for death date for seasonal analysis when the latter was missing.

Ages were used to place cases into one of three categories: reproductively immature, reproductively mature, and geriatric. For camelids, individuals were considered to be reproductively immature from newborn through 1 year old, reproductively mature from 2 to 12 years old, and geriatric at 13 years old for llamas and 12 years old for alpacas (16). Sheep and goats were considered to be reproductively immature from newborn through 1 year old, reproductively mature from 2 to 8 years old, and geriatric at 8 years old (7).

We provide a summary of cases by species, sex, age class and geographic origin, as well as monthly and annual *P. tenuis*-induced mortality rates. Rate of *P. tenuis*-induced mortality was estimated by analyzing the total number of confirmed and presumptive cases out of the total number of submissions of each species to the MNVDL. We calculated Wilson exact confidence intervals for proportions using Epitools (17). Wilson exact confidence intervals are appropriate when p is extreme; in this study many proportions were close to 0 (18). *P. tenuis*-induced mortality rates for llamas and alpacas were compared to sheep and goats using a 2 proportion z-test. A linear regression model was used to determine whether there was evidence for a trend in annual *P. tenuis*-induced mortality rates over time.

### Modeling Seasonal Effect

We were interested in determining whether there was empirical support for anecdotal trends observed for *P. tenuis* cases in livestock, such as most clinical cases (often confirmed with necropsy) occurring in late fall to early winter and large variation in *P. tenuis*-induced mortality between years (19). We also wanted to determine whether *P. tenuis*-induced mortality variability among years could be predicted by weather variables that would hypothetically influence contact with the intermediate hosts of the parasite, temperature and rainfall. We built a Generalized Circular Mixed Multilevel Model under a Bayesian framework to test these hypotheses.

First, we chose a non-linear trigonometric equation to measure the seasonality observed within a year:


(1) $$\begin{array}{l} f\left(x\right)=K+E\;\cos\left(\frac{xR\pi }{12}\right) \end{array}$$

The chosen cosine equation describes a wave-like shape, in which parameters *K, E* and *R* depict the intercept, the amplitude, and the frequency of the wave, respectively. This equation is convenient because it matches the circular nature of seasonal data [i.e., the last time unit within a year (December) is next to the first time unit (January)], as well as it holds parameters that are very easy to interpret biologically. For instance, if you consider *x* as a time unit (e.g., month) within a year, and *f(x)* as the number of *P. tenuis*-induced deaths found in that time unit, *K* would depict the yearly mean *P. tenuis*-induced mortality rate, *E* the strength of seasonality (difference between the bottom and the top of the wave), and *R* the number of peaks of infection within the year.

In our specific case, our response variable is the number of *P. tenuis*-induced deaths observed in a given number of necropsies performed monthly (*m*) throughout multiple years (*y*). So, we can assume the number of deaths follows a Binomial distribution (Equation 2):


(2) $$\begin{array}{l} {Number\;of\;deaths}_{m,y}\;\sim \;Binomial\left({Number\;of\;necropsies}_{m,y},\;p_{m,y}\right) \end{array}$$

where p_m,y_, the probability of detecting a *P. tenuis* case in one necropsy in a given month *m* of a given year *y*, can be modeled as a seasonal wave described in the Equation (1):


(3) $$\begin{array}{l} logit\left(p_{m,y}\right)=\;K+E\;\cos\left(\frac{month_{m,y}R\pi }{12}\right) \end{array}$$

Because we are also interested in the variation of parasite-induced mortality among years, we can modify Equation (3), and assume that years can present different overall mortality rates (*K*) by including the year *y* as a random intercept (“mixed effect”):


(4) $$\begin{array}{lll} logit\left(p_{m,y}\right) & = & \left(K_{0}+K_{y}\right)+E\;\cos\left(\frac{month_{m,y}R\pi }{12}\right) \end{array}$$

(5) $$\begin{array}{lll} K_{y}\; & \sim & \;Normal\left(K_{0},\;\sigma_{k_{o}}\right), \end{array}$$

where K_0_ depicts the population intercept (grand mean of the *P. tenuis*-induced mortality rate), and K_y_ is a unique *P. tenuis*-induced mortality rate estimated for each year *y* (Equation 4). Note that estimated mortality rates for each year (K_y_) are interdependent because they come from an estimated variance component (σ_ko_) centered on the population intercept (K_0_) (Equation 5).

Finally, we used a multilevel approach to look for the effect of environmental covariates that can explain the variability observed among years (K_y_):


(6) $$\begin{array}{l} K_{y}\;=\;K_{0}+\;\beta_{1}temperature_{y}+\beta_{2}rainfall_{y}, \end{array}$$

where β_1_ and β_2_ depict the linear effects of the mean seasonal (e.g., spring, summer, or fall) temperature and accumulated seasonal rainfall observed in each year *y*, respectively. Yearly environmental information was obtained from the National Weather Service Forecast Office (www.weather.gov/climate) for the state of Minnesota. Considering the solstice and the equinox dates, we considered spring to be the months from April to end of June, summer months from July to end of September, and fall months from October to end of December.

We used the Bayesian approach available in the *brms* package (20) to solve our models. We ran three Monte Carlo Markov Chains with 3,000 iterations each, burned in the first 1,500 iterations of each chain, and verified the convergence of chains and stability of the posterior distribution of estimated parameters. Priors for each fixed parameter (K_0_, *E*, β_1_, and β_2_) were set as uninformative, except for *R*, in which we limited the number of peaks to be few, positive values:


(7) $$\begin{array}{l} K_{o},\;E,\;\beta_{1},{\;\beta }_{2}\;\sim \;Normal\left(10^{5},10^{5}\right), \end{array}$$

(8) $$\begin{array}{l} R\;\sim \;Normal\left(1,\;0.5,\;minimum=0\right), \end{array}$$

Here it is worth noting that *P. tenuis* disease has a long, yet little known incubation period in small ruminants and other aberrant hosts ranging from 4 to 71 days (1, 12, 13, 21–23). Therefore, there is a delay between the actual animal infection in the field and mortality. Consequently, here we are modeling the temporal changes in the probability of detecting an infection upon mortality, rather than the actual time when the animal became infected in the field. In other words, we are modeling the temporal changes in the probability of attributing a *P. tenuis* diagnosis of mortality. Finally, we ran one model for each season (summer, spring and fall) including its respective environmental covariates. The plausibility of these three models were compared using Widely Applicable Information Criterion (WAIC).

## Results

### *P. tenuis*-induced Mortalities

**Figure 1:**
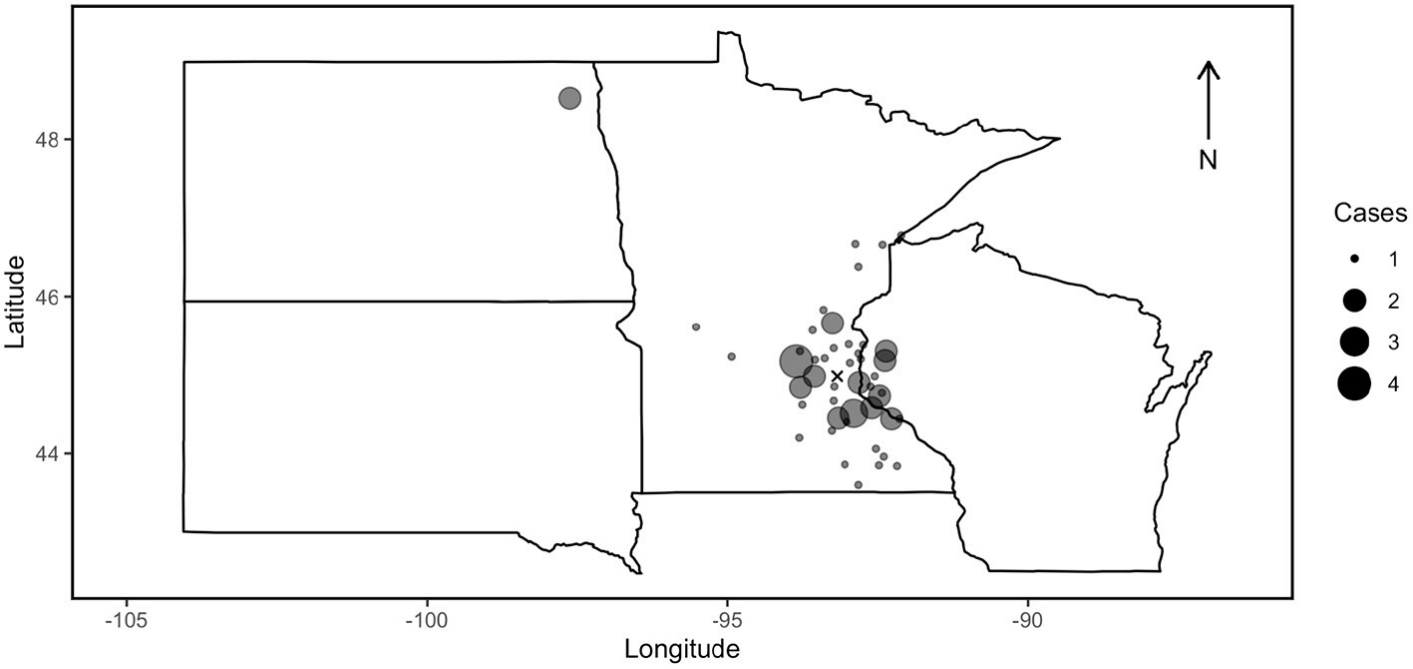
Study area. Locations of *P. tenuis* diagnosed necropsies performed by the University of Minnesota Veterinary Diagnostic Laboratory (MNVDL) on alpacas, goats, llamas and sheep between 2001 and 2019. Cases describe the number of positive cases identified at mortality at the zip code level in this timeframe. The X marks the location of the MNVDL. The same owner submitted 2 positive cases in the same year in 5 instances; otherwise individual cases were considered independent occurrences of *P. tenuis*-induced mortality.

Our underlying study population included all sheep, goats, llamas and alpacas that were voluntarily submitted to the MNVDL between January 2001 and December 2019. Of the 93 necropsy cases retrieved from the MNVDL pathology database as possible *P. tenuis* mortalities, 64 were confirmed infections based on histological evidence. The *P. tenuis*-induced mortalities diagnosed by the MNVDL originated from Minnesota (*n* = 51), North Dakota (*n* = 2) and Wisconsin (*n* = 11) (Figure 1). Females represented 53% [34/64, 95% confidence interval (CI) = 41–65%] with 20 intact, 2 pregnant and 12 with no mention of reproductive status. Males represented 33% (21/64, CI = 23–45%) of the cases, with 6 intact, 8 neutered and 7 with no mention of reproductive status. No male goats were diagnosed with *P. tenuis* infection during the study period. Nine cases had no sex listed (Supplementary Table 1). The ages of *P. tenuis* cases ranged from 6 months to 18 years old; 13 cases did not have age reported. Among the 51 animals with age reported, 18% (9/51, CI = 10–30%) were sexually immature, 71% (36/51, CI = 57–81%) were sexually mature and 10% (5/51, CI = 4–21%) were geriatric (Supplementary Table 2). There was a tendency for camelids to be sexually mature at the time of diagnosis by necropsy, which represents 30 out of the 39 necropsy cases with age reported (77%, CI = 62–87%).

The overall mortality rate associated with *P. tenuis* infection among small ruminants over the 19-year study period was 1.14% (64/5617, CI = 0.89–1.45%), with the study population being the necropsy submission pool for these species over the study time period. We observed higher *P. tenuis-*induced mortality in llamas (9.91%) and alpacas (5.33%), whereas sheep and goats each demonstrated *P. tenuis-*induced mortality levels < 1% (Table 1). *P. tenuis*-induced mortality rates for llamas and alpacas were significantly higher than those of sheep and goats (*p* < 0.001).

**Table 1:** *P. tenuis*-induced mortality by species.

**Species**	***n***	**Mortality rate (%)**	**Confidence interval (%)**
Llama	21/212	9.91	6.57–14.67
Alpaca	30/563	5.33	3.76–7.50
Goat	9/2,214	0.41	0.21–0.77
Sheep	4/2,628	0.15	0.06–0.39

*The study population consists of all necropsies performed by the University of Minnesota Veterinary Diagnostic Laboratory on alpacas, llamas, goats and sheep between 2001 and 2019*.

**Figure 2:**
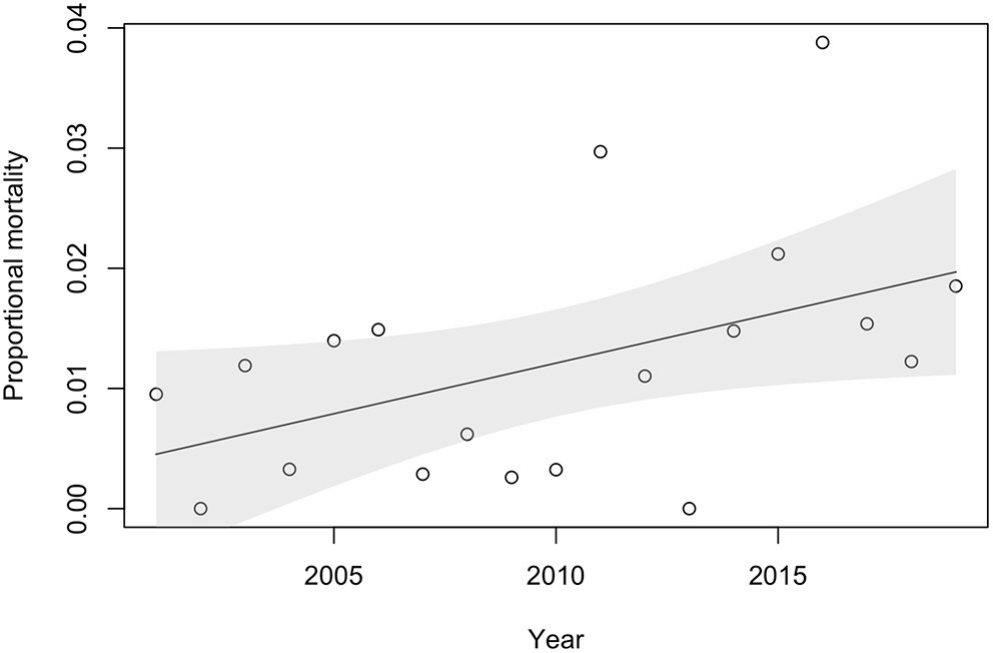
Proportional *P. tenuis-*induced mortality by year based on necropsies performed by the University of Minnesota Veterinary Diagnostic Laboratory between January 2001 and December 2019 on sheep, llamas, goats and alpacas. The solid line is the positive trend over time and the gray shaded area represents the 95% confidence interval. Data represent the number of positive *P. tenuis* necropsy cases out of the total number of necropsy cases for these species examined in the time period.

An average of 3.4 animals were diagnosed with *P. tenuis* on necropsy each year, for a mean annual mortality rate of 1.21% over the study period. However, *P. tenuis*-induced annual mortality ranged between 0% (2002 and 2013) and 3.9% (2016). We observed a significant positive trend in *P. tenuis*-induced mortality rates over time (*p* = 0.043, β = 0.0008, Figure 2).

**Figure 3:**
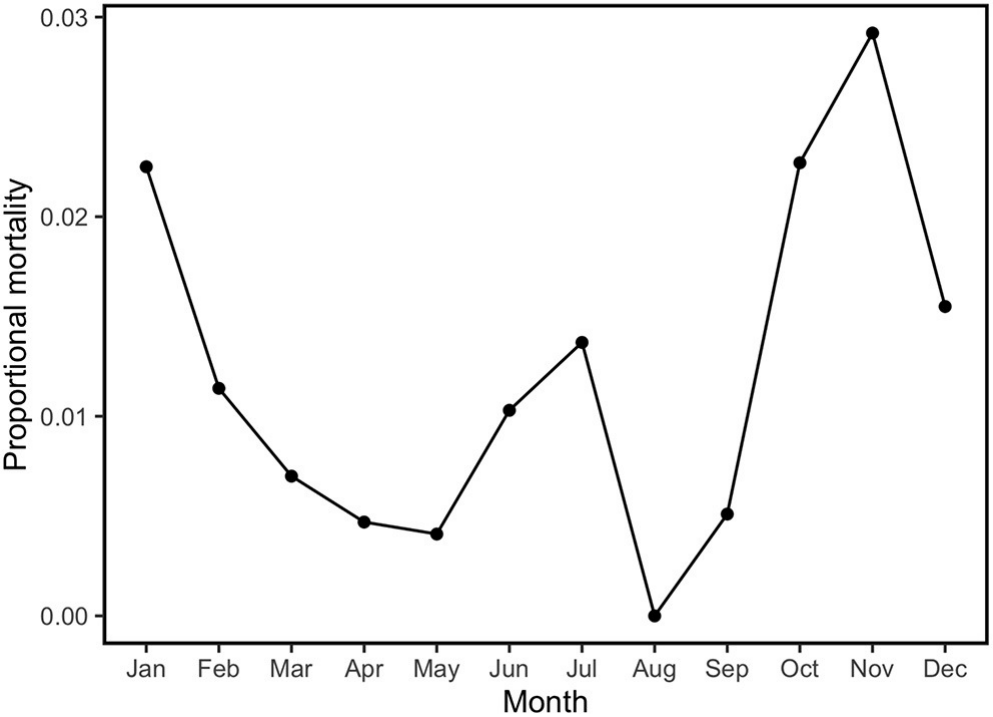
Proportional *P. tenuis*-induced mortality by month based on necropsies that came into the University of Minnesota Veterinary Diagnostic Laboratory between January 2001 and December 2019 of sheep, llamas, goats, and alpacas. Data represent the number of positive *P. tenuis* necropsy cases out of the total number of necropsy cases for these species examined in the time period.

During the study period, monthly *P. tenuis*-induced mortality rates ranged from 0 to 3% with the lowest monthly rates observed in March and April with an average mortality rate of 0.59% and late summer (August–September) with an average rate of 0.23%. Months with the highest mortality rates occurred in fall/winter (October–February) with an average incidence of 2.03%, followed by July with a mortality rate of 1.37%. Sixty-six percent of the observed mortalities were diagnosed in fall and winter (i.e., October through February; Figure 3).

### Modeling Seasonal Effects

**Figure 4:**
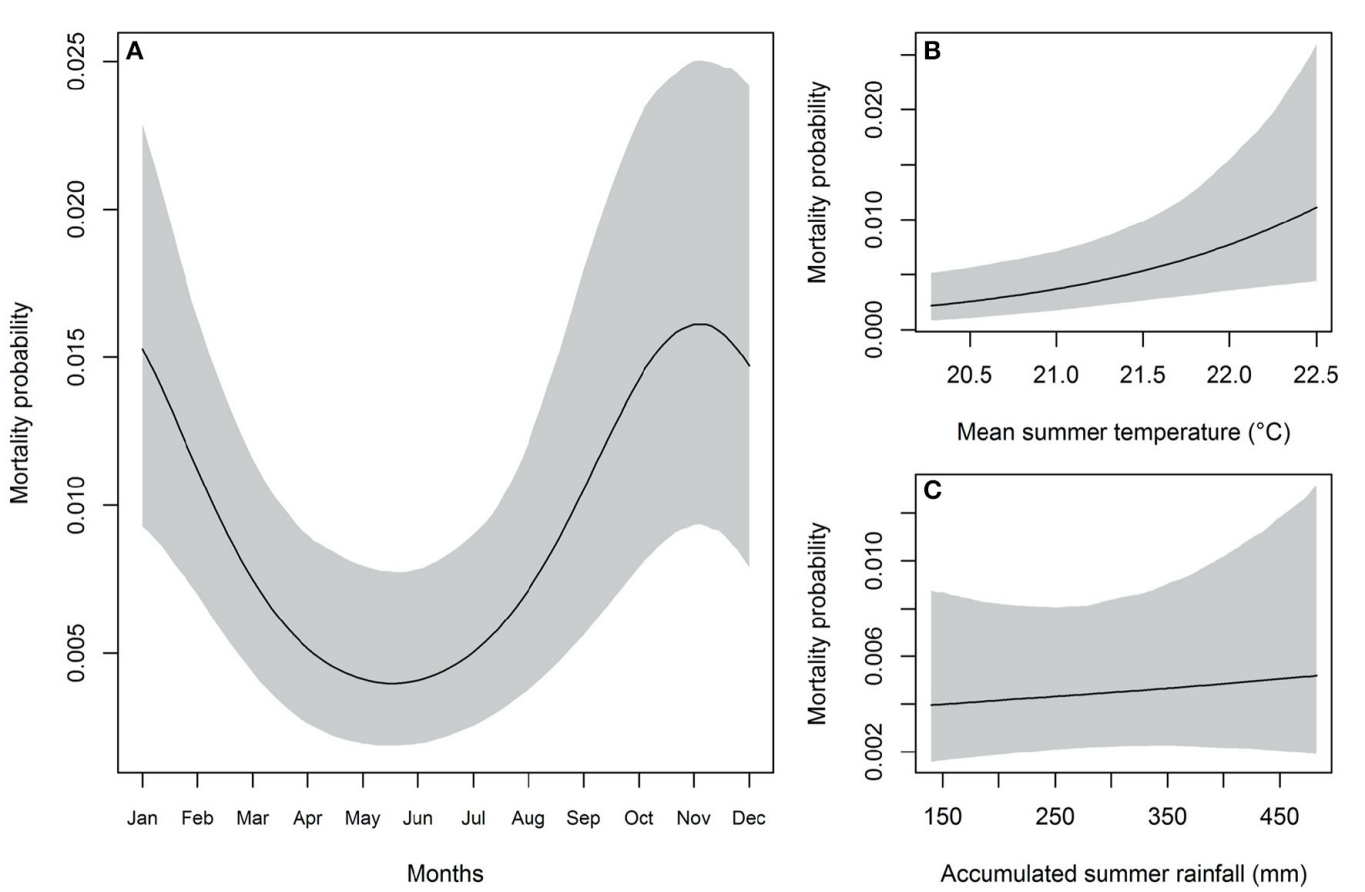
**(A)** Estimated seasonal effect of the *P. tenuis*-induced mortality probability within the year (parameters *K*_*o*_, *E and R*). Environmental effect of mean summer temperature [**(B)**; parameter β_1_] and accumulated summer rainfall [**(C)**; parameter β_2_] on the among-year variability of the probability of *P. tenuis*-induced mortality (parameter *K*_*y*_).

The Generalized Circular Mixed Multilevel Model including summer covariates was more plausible than those with spring or fall information (ΔWAIC = 4.9). Yearly temperature and rainfall were weakly correlated for summer (r = 0.23), for spring (r = −0.30), and for fall (r = 0.04). Chains converged (R_hat_ = 1 for all estimated parameters) and the posterior distribution of parameters were stable and presented a Gaussian-like distribution for the summer model (Supplementary Figure 1). According to the most plausible model (summer), the overall yearly *P. tenuis*-induced mortality rate (*K*_*o*_) was very low [mean = 1.15%, 95% credible interval (IC 95%) = 0.09–2.35%; *K*_*o*_= −11.12, IC 95% = −16.20 to −6.58] (Figure 4A). Even though the overall mortality rate was low, we detected one peak (R = 1.08, IC 95% = 0.92–1.23) seasonal effect within the year (E = 0.75, IC 95% = 0.32–1.17). Mortality probability was 3.95 times (IC 95% = 3.34–4.95) higher during October through December (top of the seasonal wave; ~1.62%) when compared to its bottom in May and June (0.048%; Figure 4A). The intra-class correlation value (ICC = 0.10, IC 95% = 0.003–0.31) indicated that about 10% of variability in monthly mortality rate was attributed to the variability among years. Furthermore, this among-year variability was positively driven by the mean summer temperature (β_1_= 0.17, IC 95% = 0.05–0.30; Figure 4B), but not by the accumulated summer rainfall (β_2_= 0.04, IC 95% = −0.13 to 0.20; Figure 4C).

## Discussion

*P. tenuis* is a potentially fatal parasite of certain livestock species, yet there is limited research into how transmission patterns and weather-related factors drive infection and mortality. We explored this using 19 years of historical necropsy records from small ruminants and camelids in Minnesota and the surrounding region. Passive surveillance and the relatively small sample size is one limitation of this study; however the almost two decades worth of data allowed key insights into this less-studied parasitic infection of small ruminants and camelids managed in the midwestern region. For instance, while overall *P. tenuis*-induced mortality was low, camelids demonstrated a higher incidence of *P. tenuis*-induced mortality than sheep or goats. We also confirmed anecdotal reports of annual and seasonal variations in *P. tenuis-*induced mortality, with the highest levels of seasonal mortality occurring October-December and annual variation in *P. tenuis*-induced mortality associated with summer temperature. While practitioners have a general understanding of the infection process, our epidemiological approach reinforces anecdotal observations and adds to the existing empirical knowledge.

The Generalized Circular Mixed Multilevel Model demonstrated just one seasonal peak from October through December where there was a 3.95 times higher probability of death due to *P. tenuis* infection in comparison to May and June. This is consistent with other reports of disease occurring in small ruminants from late summer to winter (7). Since our model was based on mortality, however, this is not the timeframe of exposure and initial infection. Literature suggests that the incubation period of *P. tenuis* in small ruminants ranges from 28 to 60 days (12, 13), and experimentally infected small ruminants demonstrated incubation periods that ranged from 4 to 71 days (13, 21–23). Thus, based on these observations, we estimate that the animals in this study likely became infected ~2 months prior to mortality, which suggests infection occurred in late summer to early fall. This is consistent with our best fitting model, which also indicated that summer temperature predicted mortality. Thus, to reduce infection risk to small ruminants here in the midwest, preventative measures should be taken in mid to late summer, when conditions contribute to higher risk.

While precipitation and humidity have more commonly been linked to *P. tenuis* prevalence (12, 24, 25), we found evidence of a positive association between summer temperature, but not precipitation, on *P. tenuis*-induced mortality in livestock. The perception of the importance of moisture for *P. tenuis* transmission is likely influenced by the well-documented importance of hydration for terrestrial gastropod intermediate hosts, leading to cessation of activity during dry conditions known as aestivation (26). However, aestivation slows but does not prevent *P. tenuis* larval development within gastropods (4). Other studies have reported positive associations between *P. tenuis* prevalence and both precipitation and temperature during the growing season (27). One mechanism for the potential importance of summer temperatures on *P. tenuis*-induced mortality is that higher temperatures may hasten *P. tenuis* larval development within gastropod intermediate hosts, which has been observed in *Parelaphostrongylus odocoilei* (28). However, there is a complex interplay between weather conditions at multiple stages of the lifecycle of *P. tenuis* that all influence risk, from L1 larvae survival, to gastropod infection, development within gastropod hosts, gastropod activity, and ungulate host survival and behavior (8). Alternatively, the relationship between *P. tenuis*-induced mortality and summer temperatures in livestock may be influenced by weather-influenced variations in livestock management. Contact with intermediate hosts could be altered if owners change their feeding practices when temperatures are higher, such as pasturing livestock in different locations or other changes in feeding practices.

A positive association between summer temperature and *P. tenuis*-induced mortalities in livestock is a worrying trend in the age of climate change. We also observed a significant increase in *P. tenuis*-induced mortalities over time during the study period. Summer temperatures have increased by 1°C in the study area between 1951 and 2012 (29), and are only expected to continue to increase in the future. If the observed trends continue, we might expect an increase in *P. tenuis*-induced livestock mortalities as the climate continues to warm.

Our observation of a higher proportion of necropsy cases showing signs of *P. tenuis* infection in llamas and alpacas compared to sheep and goats matches available information on species susceptibility. Experimental infection studies show that llamas are the most susceptible to *P. tenuis* (13, 30), followed by alpacas (23), goats (31), and sheep (32). However, other factors complicate across species comparisons, such as potential differences in the likelihood that each species will be submitted for necropsy, as well as differences in foraging behavior (that brings them into contact with infected intermediate hosts) and management.

Observational, herd-level *P. tenuis* prevalence has not been compared across these four species in a single study. We found no studies of *P. tenuis* prevalence in alpacas, though there have been reports of prevalence at the herd level of 36% for llamas (33), from 2% (6) to 59% (34) in sheep, and 0.5 to 22% in goats (5, 12). Attempts to compare herd-level prevalence observations with proportional necropsy cases attributed to *P. tenuis* are difficult for several reasons. Necropsy data do not include animals that were treated and survived infection or any that died, but were not submitted for diagnostic post-mortem examination. Indeed, at least four necropsy case reports contained observations of multiple animals in a herd displaying clinical signs of disease, but only one individual was submitted for necropsy.

The almost ubiquitous abundance of snails and white-tailed deer in the midwest, along with a lack of antemortem diagnostic testing options makes controlling *P. tenuis* infections in small ruminants and camelids very challenging for owners. While we demonstrate low *P. tenuis*-induced mortality rates in goats and sheep and moderate *P. tenuis*-induced mortality rates in llamas and alpacas using passive surveillance, the infection can be fatal without early treatment. Research has begun to genetically sequence *P. tenuis* which may eventually lead to a vaccine or better diagnostic testing options (35, 36). Other means of prevention include taller fences to keep white-tailed deer out or co-grazing with poultry as a potential way to manage gastropod numbers (9, 12, 37).

Our study provides key epidemiologic insights related to seasonality and annual variations in mortality related to temperature that can better inform owner disease prevention strategies and early detection as they manage this disease caused by *P. tenuis* in their livestock herds. Further, given the positive association of *P. tenuis*-induced mortalities with summer temperatures, more work is needed to fully understand the prevalence of infection and disease in these species, particularly as we might expect the risks of infection to increase in the midwest as climate change continues.

## Data Availability Statement

The data analyzed in this study is subject to the following licenses/restrictions: the data involves veterinary owner information confidentiality. Requests to access these datasets should be directed to kmarchetto@gmail.com.

## Ethics Statement

Ethical review and approval was not required for the animal study because the data for the study are necropsy records from the University of Minnesota Veterinary Diagnostic Lab. Written informed consent for participation was not obtained from the owners because the study is a retrospective analysis of previously collected data from a nineteen year period.

## Author Contributions

TW, AW, and KM conceived of the study. AW, KM, and CK collected the data. CK, KM, and LO-S analyzed the data. CK, KM, LO-S, and TW wrote the manuscript. CK, KM, LO-S, AW, and TW revised the manuscript. All authors contributed to the article and approved the submitted version.

## Funding

Funding for this project was provided by the Minnesota Invasive Terrestrial Plants and Pests Center through the Environment and Natural Resources Trust Fund as recommended by the Legislative-Citizen Commission on Minnesota Resources (LCCMR).

## Conflict of Interest

The authors declare that the research was conducted in the absence of any commercial or financial relationships that could be construed as a potential conflict of interest.

## Publisher's Note

All claims expressed in this article are solely those of the authors and do not necessarily represent those of their affiliated organizations, or those of the publisher, the editors and the reviewers. Any product that may be evaluated in this article, or claim that may be made by its manufacturer, is not guaranteed or endorsed by the publisher.
