# Supplementary material for: Epidemiological Investigation of Meningeal Worm-Induced Mortalities in Small Ruminants and Camelids Over a 19 Year Period
Source: Front Vet Sci. 2022 Apr 6;9:859028. doi: 10.3389/fvets.2022.859028 (PMC9020814; doi:10.3389/fvets.2022.859028)
Supplement: Supplementary file 1 [file Data_Sheet_1.docx]

Table S1. *Parelaphostrongylus tenuis*-induced mortality by species, sex and reproductive status. Based on necropsies performed by the University of Minnesota Veterinary Diagnostic lab on alpacas, llamas, goats and sheep diagnosed with *P. tenuis* between 2001 and 2019.


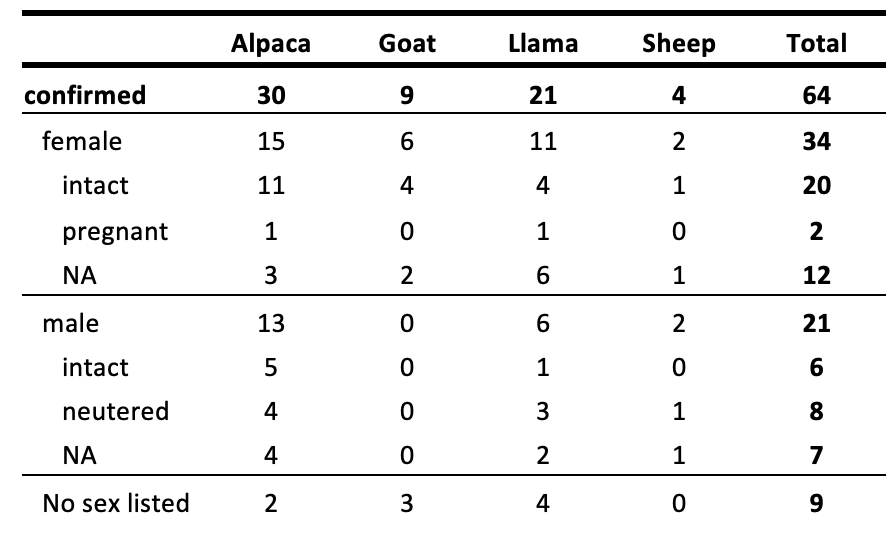


Table S2. *Parelaphostrongylus tenuis*-induced mortality by species, sex and sexual maturity (age).


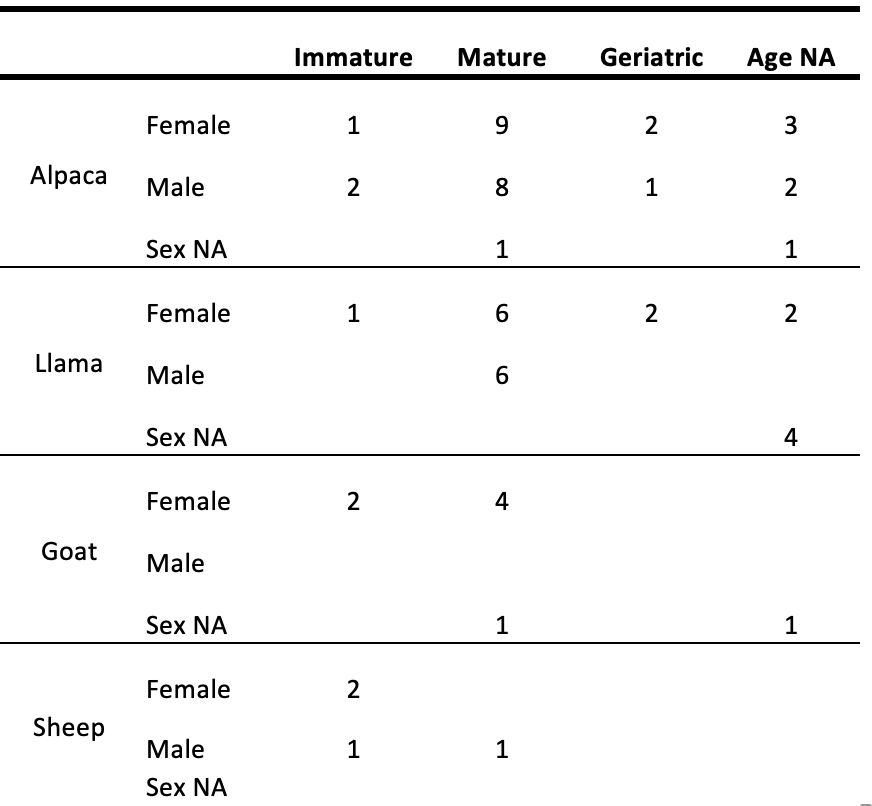


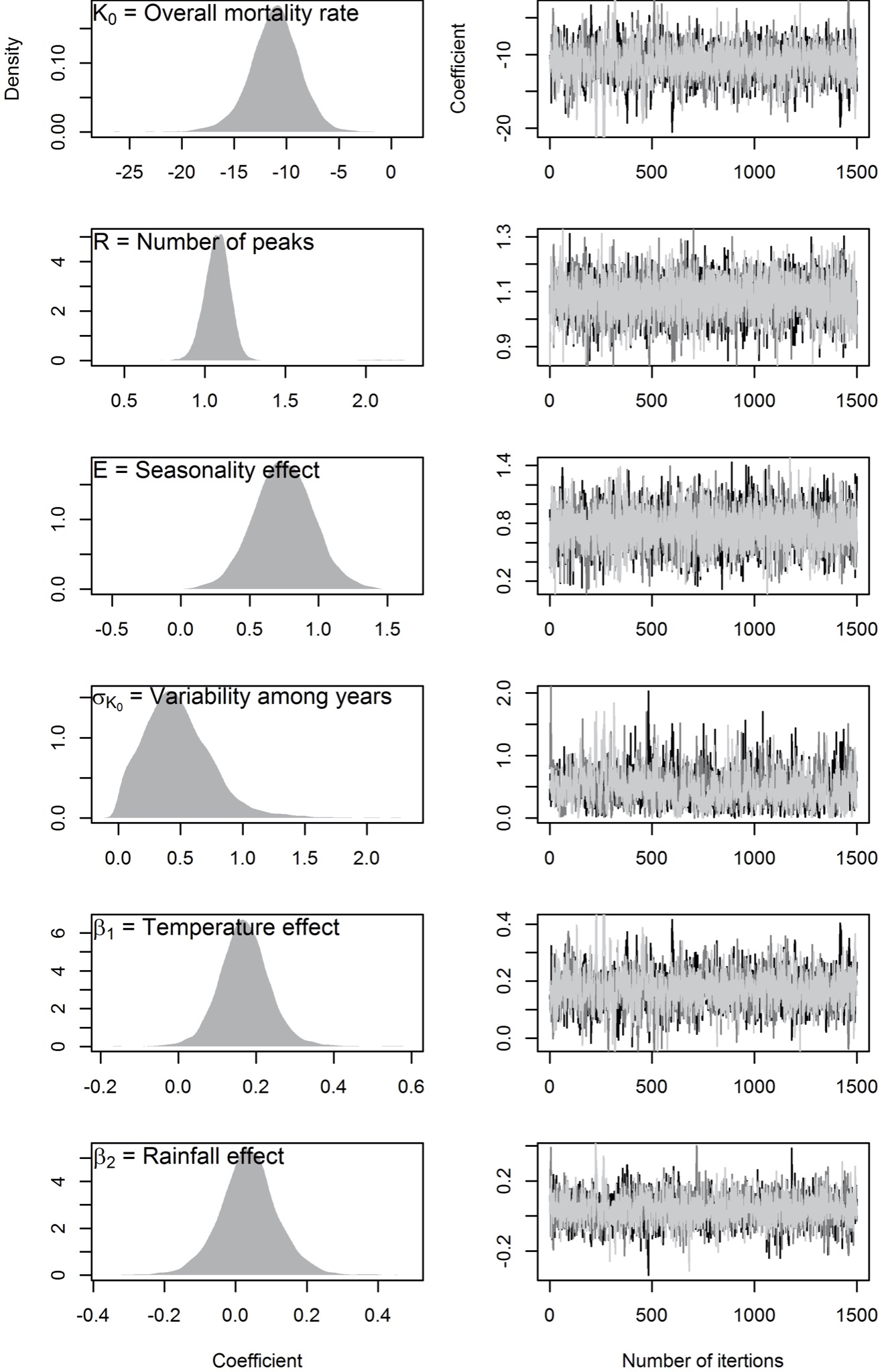


Figure S1. Posterior distribution of the estimated parameters of the three 3000-iterations chains. The first 1500 iterations of each chain were burned in. Left panels indicate the posterior distribution of each estimated parameter, while right panels indicate the convergence among the three Monte Carlo Markov Chains.
